# The Effect of Caffeine on the Velocity of Half-Squat Exercise during the Menstrual Cycle: A Randomized Controlled Trial

**DOI:** 10.3390/nu11112662

**Published:** 2019-11-04

**Authors:** Blanca Romero-Moraleda, Juan Del Coso, Jorge Gutiérrez-Hellín, Beatriz Lara

**Affiliations:** 1 Exercise Physiology Laboratory, Camilo José Cela University, 28692 Madrid, Spain; bromero@ucjc.edu (B.R.-M.); jhellin@ucjc.edu (J.G.-H.); blara@ucjc.edu (B.L.); 2 Centre for Sport Studies, Rey Juan Carlos University, Fuenlabrada, 28943 Madrid, Spain; 3 Exercise and Sport Sciences, Faculty of Health Sciences, Universidad Francisco de Vitoria, 28224 Pozuelo, Spain

**Keywords:** women, resistance exercise, exercise training, velocity, ergogenic aid, muscle function

## Abstract

Recent literature confirms the ergogenic effect of acute caffeine intake to increase muscle strength and power in men. However, the information about the effect of caffeine on muscle performance in women is uncertain and it is unknown whether its ergogenicity is similar during the menstrual cycle. The goal of this investigation was to assess the effect of acute caffeine intake on mean and peak velocity of half-squat exercise during three different phases of the menstrual cycle. Thirteen trained eumenorrheic athletes (age = 31 ± 6 years; body mass = 58.6 ± 7.8 kg) participated in a double-blind, crossover and randomized experimental trial. In the early follicular (EFP), late follicular (LFP) and mid luteal phases (MLP), participants either ingested a placebo (cellulose) or 3 mg/kg/bm of caffeine in an opaque and unidentifiable capsule. In each trial, participants performed a half-squat exercise at maximal velocity with loads equivalent to 20%, 40% 60% and 80% of one repetition maximum (1RM). In each load, mean and peak velocity were measured during the concentric phase of the exercise using a rotatory encoder. In comparison to the placebo, a two-way ANOVA showed that the ingestion of 3 mg/kg/bm of caffeine increased mean velocity at 60% 1RM in EFP (Δ = 1.4 ± 2.7%, *p* = 0.04; ES: 0.2 ± 0.2) and LFP (Δ = 5.0 ± 10.4%, *p* = 0.04; ES: 0.3 ± 0.4). No other statistical differences were found for the caffeine-placebo comparison for mean velocity, but caffeine induced an ergogenic effect of small magnitude in all of the menstrual cycle phases. These results suggest that the acute intake of 3 mg/kg/bm of caffeine induces a small effect to increase movement velocity during resistance exercise in eumenorrheic female athletes. The positive effect of caffeine was of similar magnitude in all the three phases of the menstrual cycle.

## 1. Introduction

Despite the equivocal findings of previous original investigations [1,2,3,4], emerging literature using meta-analysis suggests that acute caffeine intake is able to increase muscle strength and power [5,6]. This new information has given support to consider caffeine as an effective strategy to increase performance in resistance exercise with a relatively low prevalence of side effects when taken in the recommended doses (i.e., 3 to 9 mg per kilogram of body mass: mg/kg/bm [7]). However, most of this body of research has been carried out only on male samples. For example, in the meta-analyses by Grgic et al., about caffeine ergogenicity on muscle performance [5,6], only 9.7%–22.2% of the total sample used for these analyses were women. In fact, a detailed analysis of [5] revealed a significant increase in upper body muscle performance with caffeine in men while this effect was not present in women. Thus, caution is needed when assuming that the ergogenicity of caffeine for resistance exercise is also present in women [7]. 

Although some investigations have found an ergogenic effect of caffeine on muscle performance in women [1,8,9], this has not always been the case [10]. In general terms, it seems that the effectiveness of caffeine in increasing resistance exercise performance is lower in women than in men [11]. Sabblah et al. (2015) examined the effects of 5 mg/kg/bm of caffeine on the bench press and squat one repetition maximum (1RM) in both men and women and found there was a tendency towards an ergogenic effect of caffeine in the weight lifted in males only. Nevertheless, one common limitation of these investigations is that none of the studies controlled for the potential effects of the menstrual cycle on muscle performance [12] nor for the possible interaction of caffeine with the fluctuations of female sex hormones during the menstrual cycle [13,14]. 

Although the pharmacokinetics of acute caffeine intake are similar in the follicular, ovulatory and luteal phases [15,16], ethinylestradiol might induce an inhibition of the activity of CYP1A2, an enzyme responsible for the metabolism of caffeine [17]. In this sense, the administration of low-doses of estrogen-containing oral contraceptives reduces the rate of plasma clearance of caffeine and increases the time necessary to reach peak plasma caffeine concentration [18]. Then, the ergogenicity of caffeine to increase muscle strength might be higher in the days when the concentration of natural estrogens is higher (i.e., late follicular phase) because the serum caffeine concentration would remain longer than in the menstrual cycle phases were serum estrogen concentrations are low (i.e., menses and luteal phase) [19]. In addition, previous investigations have reported higher caffeine-induced effects on cardiovascular and subjective variables in the follicular phase than in the luteal phase [13,20]. With this background of knowledge, to date it is difficult to ascertain whether acute caffeine intake could improve muscle performance in women during resistance exercise. Furthermore, it is unknown if the potential ergogenic effect of caffeine on muscle performance is present, and of similar magnitude, during all the different phases of the menstrual cycle. Therefore, the main aim of this investigation was to determine the effect of caffeine intake on muscle performance during the early follicular, late follicular and mid-luteal phases of the menstrual cycle in eumenorrheic females.

## 2. Materials and Methods 

### 2.1. Participants

Thirteen healthy trained women volunteered to participate in this study (age = 31 ± 6 years; body mass = 58.6 ± 7.8 kg; body height = 1.66 ± 0.06 m; body fat percentage = 14.5 ± 6.5%). All of the participants were competitive athletes and fulfilled the following inclusion criteria: a) age between 18 and 40 years; b) active training (including a combination of running, cycling and swimming practice) of ~2 h/day, at least 5 days/week for the previous two months; c) low caffeine consumption (i.e., <100 mg/day); and d) steady duration of their menstrual cycle for the previous 4 months. Participants were excluded if they reported a) any type of injury within the previous six months; b) a positive smoking status; c) medication usage within the previous month; d) previous history of cardiopulmonary diseases; e) oral contraceptive use; f) allergy to caffeine; or g) any type of menstrual disorders such as dysmenorrhea, amenorrhea, or strong symptoms associated with pre-menstrual syndrome. Participants were included if they had at least six months of resistance training experience (16 ± 8 months of experience in this sample), and were familiar with the half-squat exercise. All this information was obtained from a pre-participation screening that included a medical and training history as well as a food frequency questionnaire. One week before the experiment protocol, participants were fully informed of the procedures and the risks associated with the experiment. Participants signed their informed written consent prior to participating in the investigation. The study was approved by the Camilo José Cela University Research Ethics Committee. All research protocols were in accordance with the latest version of the Declaration of Helsinki.

### 2.2. Experimental Design 

**Figure 1:**
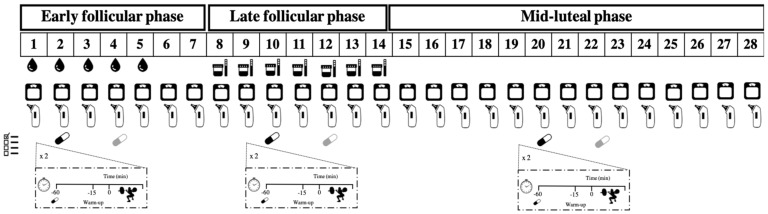
Experimental design of the investigation. 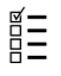 ad hoc questionnaire; 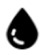 Menses; 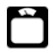 Body mass measurement; 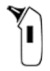 Tympanic temperature measurement; 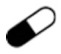 Caffeine/placebo trials; 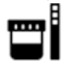 measurement of urinary peak of the luteinizing hormone; 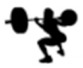 Protocol of resistance exercise.

A double-blind, placebo-controlled, crossover and randomized experimental design was used in this investigation. In each of the following three phases of the menstrual cycle: early follicular (EFP), late follicular (LFP) and the mid-luteal (MLP), each participant completed 2 experimental trials making a total of 6 identical experimental trials (Figure 1). In each trial, leg muscle performance was measured using a half-squat exercise at maximal velocity with loads equivalent to 20%, 40% 60% and 80% of one repetition maximum (1RM). In each load, mean and peak velocity were measured during the concentric phase of the exercise. During each of these three menstrual cycle phases, and in a randomized order, participants ingested an opaque and unidentifiable capsule containing either caffeine (3 mg/kg/bm; 100% purity, Bulk Powders, UK) or an inert substance as a placebo (e.g., cellulose; 100% purity, Guinama, Spain). These two trials within each phase were separated by 48 h to allow recovery, testing reproducibility, and substance elimination. The first menstrual cycle phase under investigation was randomly assigned, and a similar number of participants started in EFP (5 participants), LFP (4 participants) and MLP (4 participants). An alphanumeric code was assigned to each trial by a person independent of the study. This was done in order to double-blind the participants and researchers to the trial order and substances. Menstrual cycle phase identification was carefully conducted according to the methodological considerations raised by Janse de Jonge [21] and with the help of a period tracker application, tympanic temperature, body mass changes and assessment of the urinary peak of the luteinizing hormone. 

This image displays the protocol followed by an athlete with a 28 day menstrual cycle. After participants had recorded the regularity and length of their menstrual cycles for 4 months, caffeine (3 mg/kg/bm) or a placebo was administered in three different phases of the menstrual cycle: early follicular, late follicular and mid-luteal. Muscle performance were measured 60 min after the assigned capsule was ingested. They then measured their basal tympanic temperature, body mass, and increases in luteinizing hormone using urine test strips to determine the onset of each menstrual cycle phase. 

### 2.3. Standardizations, Familiarization and Pre-Experimental Trial

Once participants had fulfilled all the inclusion/exclusion criteria and signed the informed consent, they were encouraged to avoid nutritional supplements and sympathetic-adrenergic stimulants for the duration of the study. Participants were explicitly encouraged to avoid any nutritional source of caffeine (coffee, tea, soft and energy drinks, chocolate), and were informed about the necessity of maintaining their habitual training routines and a stable state of physical fitness during the experiment. Two weeks before the onset of the experiment, participants performed two familiarization sessions with the testing protocol in order to minimize any learning effects during the experiment. One week before the experiment, a 1RM test was performed to standardize the loads in the subsequent experimental sessions. For this 1RM measurement, participants commenced with sets of increasing loads estimated to be between 20% and 90% of 1RM, as previously described by Banyard et al. [22]. Then, the first 1RM attempt was performed with a maximum of five 1RM attempts permitted. After any successful 1RM attempt, the barbell load was increased between 0.5 and 2.5 kg until the last successful lift with a correct technique was obtained, which was categorized as 1RM (96.5 ± 17.1 kg). Two minutes of recovery were taken between 1RM attempts. On this day, participants were nude-weighed (±50 g, Radwag, Poland) in order to properly calculate caffeine dosage. The day before each trial, participants performed light, standardized training and a self-selected precompetitive diet/fluid routine was kept and recorded for replication. Participants were also required to refrain from intaking alcohol and to maintain a sleep pattern with at least 8 h of sleep the day before each trial. 

### 2.4. Experimental Protocol

Participants performed six identical experimental trials starting with the menstrual cycle phase that was randomly assigned. Trials were performed in a laboratory, in the morning (between 09:00 and 11:00) and under similar environmental conditions (22–23 °C and 60% humidity; OH1001, OH Haus, Spain). Participants arrived at the laboratory in a fed state (~3 h after their last meal). In each experimental trial, the participants were nude-weighed after voiding (Tanita BF 350, Tanita Corporation, Tokyo, Japan), and then ingested the assigned capsule with caffeine or a placebo—and rested supine for 45 min. They subsequently performed a standardized 15 min warm-up protocol that included pedaling on a cycle ergometer and a submaximal attempt on the half squat machine. Then, participants performed two attempts of the half-squat exercise with loads that represented 20%, 40%, 60% and 80% of their 1RM—measured in the pre-experimental trial. The testing was performed on a Smith Machine (Technogym, Barcelona, Spain) in which 2 vertical guides regulated the barbell movement. Participants were encouraged to produce each repetition at their maximal velocity, and they could repeat any attempt if they considered that this was not maximal. Two minutes of passive rest were allocated between the attempts with the same load and three minutes of resting between different loads. The complete range of motion for the half squat exercise consisted of lowering the body by bending the knees to a 90° angle until touching a bench with the buttocks. In this position, participants executed a maximal velocity knee extension and thus, the concentric phase of the exercise was isolated and measured. Execution technique and motivation were standardized and monitored by 2 experienced researchers for reliability of the experimental conditions. In each attempt, barbell displacement in the concentric phase of the movement was recorded with a rotatory encoder and associated software (Isocontrol, EV-Pro, Spain) and mean and peak velocity (in m/s) were measured. The attempt with the highest barbell displacement velocity in each load was used for statistical analysis. With this information, the estimated 1RM was calculated [23] in all phases to ensure that 1RM remained unchanged throughout the experiment (EFP: 97.0 ± 23.2 kg; LFP: 98.5 ± 18.1 kg; MLP: 98.1 ± 22.2 kg). 

### 2.5. Determination of Menstrual Cycle Phase

The EFP, LFP and MLP phases were selected for investigation because they represent main events occurring during the menstrual cycle (i.e., menses, pre-ovulation and peak progesterone concentration, respectively). The duration of the menstrual cycle and the onset of each phase were accurately determined by using (a) a period tracker application; (b) measurement of basal tympanic temperature and body mass changes; and (c) assessment of the urinary peak of the luteinizing hormone, following established recommendations [21]. The duration of each participant’s menstrual cycle was recorded for a minimum of 4 months prior to the onset of the experiment for a valid characterization of length. This information was obtained using a mobile application (Mycalendar®, Period-tracker, Hong Kong, China) together with a menstruation diary, which included the date of menses, length of menses, and discomfort in the days preceding and during the menses. All participants had a regular menstrual cycle for the four months previous to the experiment (27 ± 2 days, range = 24–31 days) and were considered as eumenorrheic. During the familiarization period, participants were trained on how to measure their own basal tympanic temperature and to obtain valid body mass measurements. A digital thermometer (model HDT8208C, Nursal Ear Thermometer, Dongguan, China) and a digital scale (BT200, Daga, Barcelona, Spain) were provided for each participant to obtain data every morning immediately after waking up. Participants obtained these data for one complete menstrual cycle, starting with the phase randomly allocated (tympanic temperature; EFP: 36.34 ± 0.42; LFP: 36.43 ± 0.62; MLP: 36.42 ± 0.47 °C, body mass; EFP: 58.86 ± 9.28; LFP: 58.89 ± 9.14; MLP: 59.03 ± 9.11 kg). In addition, participants were supplied with 7 reactive test strips (One Step Ovulation LH Test Strip; CVS Health, Woonsocket, RI, US) to assess any increase in the luteinizing hormone in the first-morning urine sample. With all this information, the following events were used to determine the onset of each phase, as follows: EFP was indicated by the onset of menses; LFP was indicated by a positive test for urinary luteinizing hormone; MLP was determined to be between 70% and 75% of the individual menstrual cycle length (i.e., from the 20th to 22th day of the menstrual cycle for a regular cycle of 28 days [21]). All these protocols helped to align the participants’ cycles and therefore, despite different cycle lengths, participants performed the testing in the same cycle phases.

### 2.6. Statistical Analysis

Data were collected as previously indicated and the results of each test were blindly introduced into the statistical package SPSS v 20.0 (IBM company, New York City, NY, US) for later analysis. Normality was tested for each variable with the Shapiro–Wilk test. All included variables in this investigation presented a normal distribution (*P* > 0.05) and parametric statistics were used to determine the ergogenicity of caffeine. The caffeine-placebo differences in mean and peak velocity were identified using a two-way ANOVA with repeated measures (treatment × load). After a significant *F* test, differences among means were identified using the Bonferroni post hoc procedure. The significance level was set at *P* ≤ 0.05. The results are presented as means ± SD. To improve the identification of meaningful differences, the effect size was also calculated in all caffeine-placebo pairwise comparisons to allow a magnitude-based inference approach [24]. The effect-size statistic ±90% confidence intervals (CI) was used on log transformed data to reduce bias due to non-uniformity of error. The smallest significant standardized effect threshold was set as 0.2. Ranges of likelihood <1% indicated almost certainly no chances of change; 1% to 5%, very unlikely; 5% to 25%, unlikely; 25% to 75%, possible; 75% to 95%, likely; 95% to 99%, very likely; >99%, most likely. Differences were rated as unclear when likelihood exceeded >5% in both positive/negative directions. Effect sizes were interpreted according to the following ranges: <0.2, trivial; 0.2–0.6, small; 0.6–1.2, moderate; 1.2–2.0, large; 2.0–4.0, very large and; >4.0, extremely large [24].

## 3. Results

**Figure 2:**
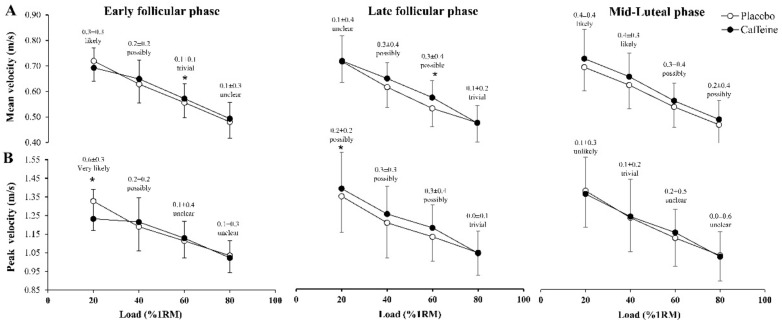
Changes induced with the ingestion of 3 mg/kg/bm of caffeine on mean velocity (**A**) and peak velocity (**B**) during the concentric phase of the Smith machine half-squat exercise of increasing loads (20%, 40%, 60% and 80% of one repetition maximum; 1RM) in each phase of the menstrual cycle. Data are mean ± standard deviation from 13 eumenorrheic athletes. The information over the data corresponds to the caffeine-placebo effect size statistic ±90% confidence intervals and magnitude base inference of this comparison. (*) Caffeine different from placebo within the same menstrual cycle phase at *P* < 0.05.

Figure 2 displays mean and peak velocity differences between caffeine and the placebo for all the loads under investigation. In comparison to the placebo, the two-way ANOVA showed that the ingestion of 3 mg/kg/bm of caffeine increased mean velocity at 60% 1RM in EFP (Δ = 1.4 ± 2.7%, *P* = 0.04) and LFP (Δ = 5.0 ± 10.4%, *P* = 0.04). No other differences were identified with the two-way ANOVA in mean or peak velocity. However, the magnitude-based inference approach showed that, in EFP, mean velocity was likely higher at 20% 1RM (Δ = 2.9 ± 4.0%, chance% as positive/trivial/negative = 55/45/0%) with placebo than with caffeine. In EFP, mean velocity was possibly higher at 40% 1RM (Δ = 3.1 ± 5.7%; 55/44/1%) with caffeine than with the placebo (Figure 2, panel A). In LFP, mean velocity was possibly higher at 40% 1RM with caffeine than with the placebo (Δ = 3.7 ± 8.7%; 63/35/2%). In MLP, mean velocity was likely higher at 20% (Δ = 5.4 ± 8.7%; 85/14/1%), and 40% 1RM (Δ = 6.1 ± 9.1%, 85/15/0%) and possibly higher at 60% (Δ = 5.3 ± 12.1%; 70/28/2%), and 80% 1RM (Δ = 4.7 ± 14.7%; 54/43/3%) with caffeine than with the placebo. 

For peak velocity, it was very likely that this variable was higher with placebo at 20% 1RM (Δ = 3.1 ± 5.7%, 55/44/1%) and possibly higher with caffeine at 40% 1RM (Δ = 3.1 ± 5.7%; 55/44/1%) in the EFP (Figure 2, panel B). Caffeine induced possible ergogenic effects on peak velocity at 20% (Δ = 3.1 ± 4.3%, 53/47/0%), 40% (Δ = 3.9 ± 7.9%, 63/35/1%), and 60% 1RM (Δ = 2.8 ± 7.7%, 60/36/4%) in the LFP with no trivial or unclear effects in the MLP.

## 4. Discussion

The current body of evidence has found that acute caffeine intake (i.e., 5–6 mg/kg/bm) is able to increase muscle strength and power in women [1,8,9,10], although the ergogenic effect of this substance was small in all these investigations. However, previous research protocols on this topic did not consider the menstrual cycle phase in which the caffeine ergogenicity was found, despite the potential interaction between female sex hormones and caffeine [13,14]. To the authors’ knowledge, this is the first study to directly compare the ergogenic response to caffeine on resistance exercise performance during the different phases of the menstrual cycle. Using a repeated-measures design in which the onset of the menstrual cycle phase was carefully delimited, caffeine-placebo comparisons were made in the early follicular, late follicular and mid-luteal phases while muscle performance was measured during a half squat force-velocity relationship. By using a traditional statistical approach, the two-way ANOVA revealed only subtle ergogenic effects of caffeine on mean and peak velocity in the half squat exercise. However, the magnitude-based inference approach indicated that caffeine was able to produce small ergogenic effects on mean and peak velocity at several loads (Figure 2) with a tendency to move the force-velocity relationship upwards in all phases of the menstrual cycle. Overall, the magnitude of these effects was comparable in all the three menstrual cycle phases under investigation. Taken together, these data suggest that caffeine might have a potential to enhance maximal velocity of movement in half-squat exercise. Although this effect was equally present during the menstrual cycle, the effect was catalogued as of small magnitude.

The concentration of the main female sex hormones fluctuates during different phases of the menstrual cycle provoking changes in physiological functions and performance [25,26,27]. The ovarian hormones estrogen and progesterone provoke opposing physiological functions—while estrogen is a hormone with a purported anabolic function, progesterone has been related to catabolic pathways [21,28]. For this reason, it has been speculated that muscle performance and muscle adaptations might be favored when estrogen concentration is high and progesterone is low (i.e., follicular phase). In fact, although muscle performance seems unaffected during the menstrual cycle [29], concentrating most of the resistance training in the follicular phase induces greater changes in muscle strength and hypertrophy compared to concentrating resistance training in the luteal phase [30]. In addition, the intake of 2 mg/kg/bm of caffeine produces greater caffeine-induced cardiovascular and mood changes in the follicular vs. the luteal phase [13,20]. Together, these effects might indicate that acute caffeine intake will produce a higher caffeine ergogenicity in the follicular phase. 

Interestingly, this speculation was not confirmed by our data because caffeine presented a similar ergogenic effect to increase mean velocity in the early follicular, late follicular and mid luteal phases (Figure 2). Caffeine produced a negative effect on peak velocity at 20% 1RM in the early follicular phase of the menstrual cycle. However, this negative effect was not found in the remaining loads of this menstrual cycle phase nor in peak velocity values of the late follicular and mid luteal phases. In the author’s opinion, this lack of effect of acute caffeine intake at 20% 1RM in the early follicular phase is anecdotical and does not alter the overall positive effect of caffeine to increase velocity during half-squat exercise (Figure 2). Caffeine produced this positive effect in the mid-luteal phase despite the probable high serum concentration of progesterone at this time of the menstrual cycle [15]. Although we did not assess serum caffeine concentrations, it is presumable that the stable caffeine metabolism during the menstrual cycle in these women who were not taken oral contraceptives [15,16] produced comparable serum caffeine concentrations in the early follicular, late follicular and mid luteal phases that promoted comparable ergogenicity for muscle performance. This finding is novel and reflects the high potential capacity of acute caffeine intake to produce increases in muscle performance in women, as previously found in other exercise and sport disciplines [31,32,33]. In this case, these data are novel because suggests that the magnitude of the caffeine ergogenic effect is comparable across the menstrual cycle. 

Nevertheless, it is very important to take into account the individual responses during the menstrual cycle. In the current investigation, eumenorrheic women with no menstrual disorders were selected as the study sample to avoid the possible effects of these symptoms on the results of the pairwise caffeine-placebo comparisons. However, there is a high percentage of athletes who report premenstrual symptoms that might ultimately decrease performance [34]. Unfortunately, the results of this investigation cannot be used to ascertain whether caffeine might be used to avoid or to reduce the performance detriments produced by any menstrual disorder and further investigation about the effects of caffeine in these populations is warranted. The ergogenic effect of caffeine on muscle performance in women taking oral contraceptives should also be investigated because ethinylestradiol, one of the substances included in contraceptive pills, decreases caffeine metabolism [17]. 

There are several limitations to this experiment that should be mentioned and discussed to understand its scope. Firstly, to determine the onset of the menstrual cycle phases there was no measure of the concentration and/or quantity of female steroids hormones. However, we used a menstrual period tracker application, and measured changes in tympanic temperature and body mass. In addition, we also used luteinizing hormone urine test strips, as previously recommended [12,35]. Secondly, although the participants who underwent this protocol had at least six months of resistance training experience, they had no experience in velocity-based training. Lastly, we only used a dose of 3 mg/kg/bm of caffeine which is lower than the 5–6 mg/kg/bm used in previous investigations on caffeine effects on muscle performance. In addition, we selected low caffeine users while it is possible that higher doses are necessary to find an ergogenic effect of caffeine in women habituated to caffeine intake, as this has been demonstrated in male athletes [36]. Thus, it is possible that the magnitude of the ergogenic effect on mean and peak velocity found in this investigation was affected by the dose and the lack of tolerance to this drug.

## 5. Conclusions

In summary, the pre-exercise ingestion of 3 mg/kg/bm of caffeine increased, to a similar extent, mean and peak velocity in the half squat exercise at increasing loads in the early follicular, late follicular, and mid luteal phases of eumenorrheic trained athletes. Thus, in eumenorrheic women, caffeine might have the potential of increasing muscle performance during the menstrual cycle, although 3 mg/kg/bm would produce an effect of small magnitude. The outcomes of this investigation suggest that eumenorrheic female athletes might use acute caffeine intake to increase movement velocity during resistance training routines. The use of caffeine might be used to increase maximal strength values on different strength-based exercises [37]. In addition, it has been recently found that resistance training performed at fast movement velocities offers superior muscular strength gains than resistance training with slow-to-moderate velocities [38]. Thus, the use of caffeine before resistance training might be effective to enhance muscle adaptation derived from long-term strength training, although such hypothesis deserves further confirmation. In this sense, caffeine ergogenicity for resistance exercise can be equally obtained in all phases of the menstrual cycle and then, the supplementation with caffeine can be used to design strength training programs without any interference with athletes’ menstrual cycle.
